# Cost-utility analysis of erenumab preventive treatment for chronic migraine in China

**DOI:** 10.3389/fpubh.2026.1878335

**Published:** 2026-09-21

**Authors:** Lijiang Wang, Menglu Zhu, Qingqing Hu, Shengying Lou, Miaolian Wu, Zhengqiang Hu

**Affiliations:** 1 Department of Pharmacy, The Fourth Affiliated Hospital of School of Medicine, and International School of Medicine, International Institutes of Medicine, Zhejiang University, Yiwu, China; 2 Research Center for Clinical Pharmacy, Zhejiang University, Hangzhou, Zhejiang, China; 3 Department of Pharmacy, The Children’s Hospital, Zhejiang University School of Medicine, National Clinical Research Center for Child Health, Hangzhou, Zhejiang, China

**Keywords:** China, chronic migraine, cost-utility analysis, erenumab, microsimulation, pharmacoeconomic evaluation

## Abstract

**Objective:**

This study aimed to evaluate the cost-utility of erenumab preventive treatment compared to no preventive treatment in adults with chronic migraine in China.

**Methods:**

An individual-level microsimulation model was developed from the perspective of the Chinese health system. The model compared erenumab 70 mg every 4 weeks with no preventive pharmacotherapy plus usual acute and migraine-related medical care. The base-case time horizon was 52 weeks, with 13 cycles of 4 weeks each. Clinical inputs were mainly derived from the DRAGON trial, which included Asian adults with chronic migraine. Week 12 monthly migraine days (MMDs) distributions were calibrated to reproduce the reported mean MMD and ≥50% MMD responder rate. Post-week 12 outcomes in the erenumab arm were extrapolated at the patient level according to treatment status and discontinuation. Costs included erenumab acquisition, acute medications, and migraine-related non-drug medical services. Outcomes included total costs, quality-adjusted life years (QALYs), the incremental cost-effectiveness ratio (ICER), and incremental net monetary benefit (INMB). One-way sensitivity analysis, probabilistic sensitivity analysis, a 12-week-horizon scenario analysis, and threshold price analysis were conducted.

**Results:**

In the base case, erenumab preventive treatment generated 0.572 QALYs at a total cost of CNY 45,810.21, whereas no preventive treatment generated 0.557 QALYs at a total cost of CNY 4,281.59. The incremental cost was CNY 41,528.62, and the incremental QALY gain was 0.015. The ICER was CNY 2,830,387.52 per QALY gained. The INMB was CNY − 40,066.30 and CNY − 37,141.64 at willingness-to-pay (WTP) thresholds of 1× and 3× Gross Domestic Product (GDP) per capita, respectively. Sensitivity analyses and the 12-week scenario analysis supported the base-case conclusion. Threshold price analysis showed that erenumab would need to decrease to CNY 141.82 and CNY 386.81 per 70-mg box to be cost-effective at WTP thresholds of 1× and 3× GDP per capita, respectively.

**Conclusion:**

From the Chinese health system perspective, erenumab as preventive treatment provides modest health gains for adults with chronic migraine but is unlikely to be cost-effective at its current price.

## 1 Introduction

Migraine is a recurrent neurological disorder that can cause pain, disability, and reduced quality of life. Chronic migraine is a more severe form of migraine and is usually defined as headache occurring on at least 15 days per month for more than 3 months, with migraine features present on at least 8 days per month (1). Patients with chronic migraine often experience more frequent attacks, greater functional impairment, and higher healthcare utilization than patients with episodic migraine. In addition to clinical symptoms, chronic migraine can generate substantial economic burden through repeated healthcare utilization and impaired daily functioning (2, 3).

Recent Global Burden of Disease (GBD) evidence continues to identify migraine as one of the leading contributors to neurological disability worldwide, and China-specific GBD analyses suggest that headache disorders remain highly prevalent in China, with a large absolute number of affected individuals and measurable disability burden (4, 5).

Preventive treatment aims to reduce the frequency and severity of future migraine attacks. Traditional preventive treatments include beta blockers, calcium channel blockers, antiepileptic drugs, and antidepressants (6, 7). Their use may be limited by inadequate responses, poor tolerability, contraindications, or poor long-term persistence. These limitations have created a need for preventive options with migraine-specific mechanisms and better long-term tolerability. Recent international guidelines have increasingly recognized calcitonin gene-related peptide (CGRP) pathway therapies as established, migraine-specific preventive options. The American Headache Society position statement recommends CGRP-targeted therapies as a first-line approach to migraine prevention, while the European Headache Federation guideline recommends monoclonal antibodies targeting the CGRP pathway for migraine prevention because of their efficacy and safety profile (8, 9). Evidence summaries prepared by the International Headache Society also support the efficacy of CGRP monoclonal antibodies in preventing episodic and chronic migraine (10).

Erenumab is a monoclonal antibody targeting the CGRP receptor. Randomized, placebo-controlled evidence from international chronic migraine populations showed that erenumab reduced monthly migraine days (MMDs) and acute migraine-specific medication days compared to placebo (11). In clinically relevant subgroups, including chronic migraine patients with medication overuse, erenumab was also associated with greater reductions in MMDs and acute migraine-specific medication days compared to placebo (12). In the DRAGON phase 3 trial involving Asian adults with chronic migraine, erenumab 70 mg once monthly reduced MMDs and improved the ≥50% MMD responder rate compared to placebo over 12 weeks (13). A *post hoc* analysis also suggested that erenumab increased reversion from chronic migraine to episodic migraine (14).

Clinical benefit alone does not establish value for money. Erenumab has a high acquisition cost, and the economic value of reducing MMDs depends on the drug price, health utility gains, changes in acute medication use, and the perspective used for costing. As acquisition prices, patterns of acute medication use, healthcare utilization, and willingness-to-pay thresholds differ across settings, economic results from other countries may not be directly transferable to China. Evidence for the cost-utility of erenumab for chronic migraine in China remains limited. Previous health technology assessments and economic evaluations from other settings indicate that the cost-effectiveness of erenumab is highly sensitive to drug price, population selection, stopping rules, comparator choice, and whether productivity losses are included (15–18).

This study, therefore, evaluated the cost-utility of erenumab preventive treatment compared to no preventive treatment in Chinese adults with chronic migraine, using an individual-level microsimulation model informed by Asian clinical evidence and China-specific cost inputs.

## 2 Methods

### 2.1 Study design and perspective

We conducted a cost-utility analysis using an individual-level microsimulation model. The target population was adults with chronic migraine. The analysis was conducted from a Chinese health system perspective and included only direct medical costs.

The model compares two strategies:

(1) Erenumab preventive treatment: Erenumab 70 mg every 4 weeks, with acute medications and migraine-related non-drug medical services allowed.
(2) No preventive treatment: No erenumab or other preventive treatment in the model, along with acute medications and migraine-related non-drug medical services allowed.

In this study, “no preventive treatment” refers to the absence of erenumab or other preventive pharmacotherapy in the model and does not imply an absence of migraine care. Patients in this strategy were assumed to continue receiving acute medications and migraine-related non-drug medical services when needed. Its short-term clinical trajectory was informed by the placebo arm of DRAGON, which reflected placebo plus background care within the trial context. This comparator was selected because the pivotal Asian evidence directly compared erenumab with placebo and because head-to-head evidence against conventional preventive therapies in Chinese adults with chronic migraine was not available. Therefore, the results should be interpreted as the incremental value of adding erenumab preventive treatment to usual acute/rescue care, rather than as a comparison with all available preventive treatment options.

The base-case time horizon was 52 weeks. The model used 13 cycles, each lasting 4 weeks. As the time horizon was 1 year, costs and quality-adjusted life years (QALYs) were not discounted. Costs were expressed in 2025 Chinese yuan (CNY). The analysis was conducted in accordance with CHEERS 2022 and the China Guidelines for Pharmacoeconomic Evaluations (19, 20).

The overall model structure is shown in Figure 1.

**Figure 1:**
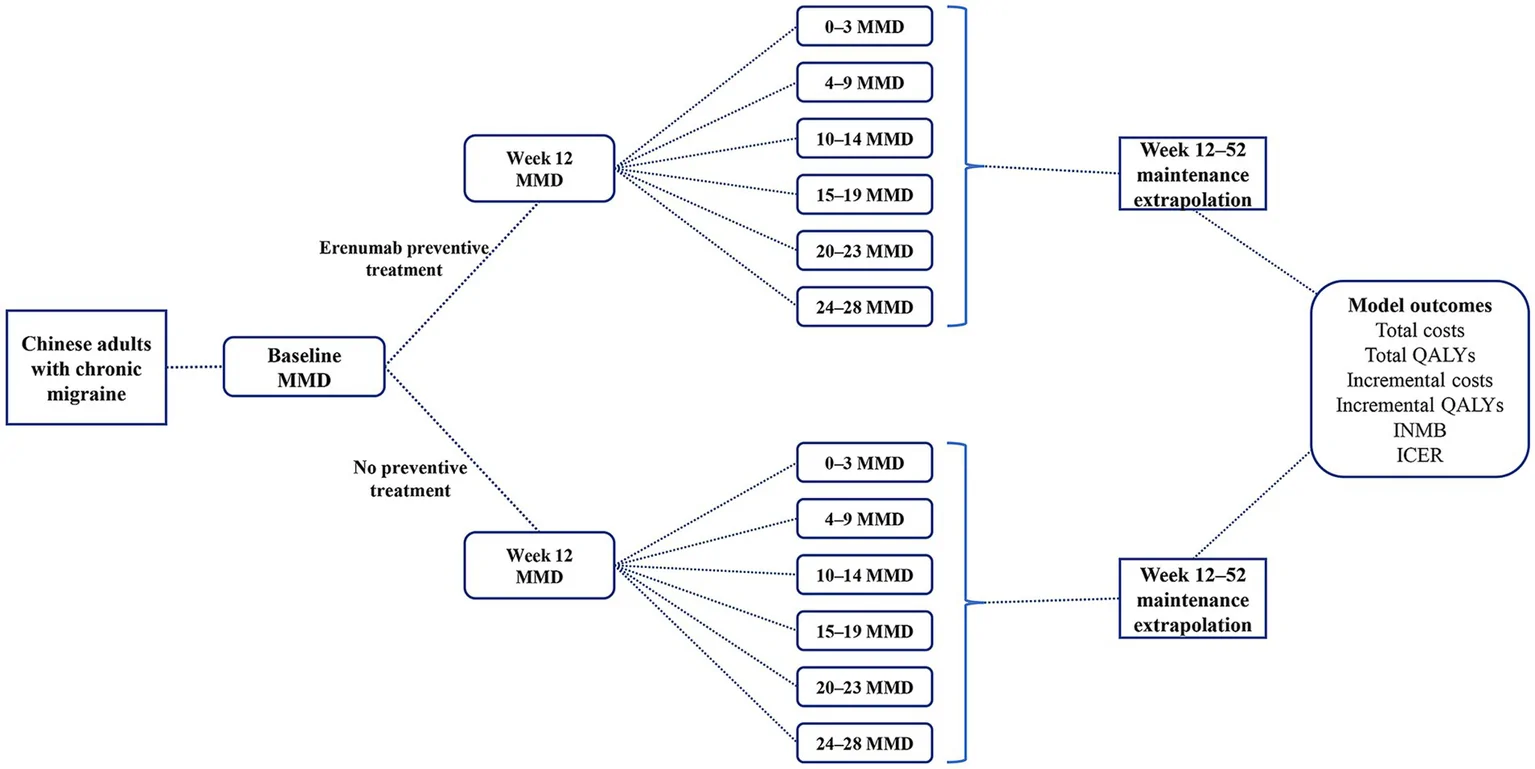
Structure of the microsimulation model. The model compared erenumab preventive treatment with no preventive treatment in adults with chronic migraine in China. The baseline MMD was sampled from the same distribution for both strategies. Week 12 MMD distributions were calibrated separately using the Week 12 mean MMD and the ≥50% MMD responder rate as primary targets. Continuous MMD values were mapped to MMD health states for utility assignment. After Week 12, the no preventive treatment arm maintained its Week 12 clinical level, whereas outcomes in the erenumab arm were extrapolated at the patient level according to treatment status, discontinuation reason, and post-discontinuation waning. MMD, monthly migraine day; QALY, quality-adjusted life year; ICER, incremental cost-effectiveness ratio; INMB, incremental net monetary benefit.

### 2.2 Model structure

The MMD was the main clinical state variable. The same baseline MMD distribution was used for both strategies. Week 12 MMD distributions were then calibrated separately for erenumab preventive treatment and no preventive treatment. Continuous MMD values were mapped to MMD health states to assign utility.

The MMD health states were defined as 0–3, 4–9, 10–14, 15–19, 20–23, and 24–28 MMDs per 4 weeks. For continuous MMD values, these were implemented as 0 ≤ MMD < 4, 4 ≤ MMD < 10, 10 ≤ MMD < 15, 15 ≤ MMD < 20, 20 ≤ MMD < 24, and 24 ≤ MMD ≤ 28.

Cycle QALYs were calculated as the mean utility multiplied by 4/52. Total QALYs were calculated as the sum of cycle QALYs across all cycles. Mortality was not modeled because the time horizon was short and neither strategy was expected to affect mortality.

### 2.3 Clinical inputs

Clinical inputs are summarized in Table 1. The baseline MMD was sampled from a truncated normal distribution bounded between 8 and 28 days per 4 weeks, with a target post-truncation mean of 19.2 and a standard deviation of 5.4. The simulated baseline distribution is shown in Supplementary Figure 1.

**Table 1:** Clinical inputs.

Parameter	Base value	Range	Source
Time horizon	52 weeks	—	–
Cycle length	4 weeks	—	–
Baseline MMD, mean	19.2	—	(13)
Baseline MMD, SD	5.4	—	(13)
Baseline MMD bounds	8–28	—	(13)
Week 12 MMD change, erenumab preventive treatment	−8.2	−9.84 to −6.56	(13)
Week 12 MMD change, no preventive treatment	−6.6	−7.92 to −5.28	(13)
Baseline acute medication days	14.3	—	(13)
Week 12 acute medication days, erenumab preventive treatment	8.96	—	(13)
Week 12 acute medication days, no preventive treatment	9.64	—	(13)
12-week all-cause treatment discontinuation probability	2.15%	1.72–2.58%	(13)
52-week open-label treatment discontinuation probability	22.80%	18.24–27.36%	(21)
Conditional proportion of treatment discontinuations due to lack of efficacy	28.07%	22.46–33.68%	(21)
Retained positive incremental benefit after other-reason discontinuation, cycles 1/2/3	0.91/0.64/0.45	—	(22)

MMD, monthly migraine day.

Short-term efficacy inputs were mainly taken from DRAGON (13). Week 12 MMD changes were −8.2 days for erenumab preventive treatment and −6.6 days for no preventive treatment. Baseline acute medication days were 14.3 days per 4 weeks. Week 12 acute medication days were 8.96 and 9.64 for erenumab preventive treatment and no preventive treatment, respectively.

### 2.4 Week 12 MMD calibration

Individual-level DRAGON data were not available. Therefore, Week 12 continuous MMD distributions were generated through calibration to reproduce the main aggregate outcomes reported in the trial. The primary calibration targets were the Week 12 mean MMD and the ≥50% MMD responder rate in each strategy. The proportion of patients with an MMD value <15 was included as a secondary soft target because the related DRAGON *post hoc* endpoint was based on headache days rather than MMDs (14). For this reason, an MMD value <15 was used to guide the shape of the calibrated distribution but it was not required to match exactly. Calibration targets and model fit are summarized in Supplementary Table 1.

Week 12 continuous MMD values were generated using a two-component truncated normal mixture distribution. The generated marginal distribution was assigned to simulated baseline patients using a rank-based approach to reproduce the observed ≥50% responder rate. The resulting distributions are model-generated summaries that are consistent with the available aggregate evidence, rather than reconstructed individual-level DRAGON data. The calibrated Week 12 continuous MMD distributions are shown in Supplementary Figure 2. As exact agreement across all calibration summaries was not required for the secondary MMD < 15 target, we additionally examined the robustness of economic outcomes to the trade-off between the primary MMD-based targets and this secondary distributional target.

### 2.5 Extrapolation from weeks 12 to 52

The DRAGON double-blind period provided direct comparative evidence up to Week 12. For the 52-week base case, assumptions were required to describe clinical outcomes after Week 12. In the no preventive treatment arm, Week 12 MMDs and acute medication use were assumed to remain at their Week 12 levels through Week 52.

In the erenumab arm, post-Week 12 outcomes were extrapolated at the patient level. Patients who remained on treatment retained their Week 12 MMD and acute medication use levels. Maintenance treatment discontinuation was informed by the 52-week open-label extension of erenumab, in which 139 of 609 patients discontinued open-label treatment (21). This probability was converted into a constant 4-week probability and applied after Week 12 to patients who remained on treatment.

Among these treatment discontinuations, 39 of 139 were due to lack of efficacy. Each simulated discontinuation was therefore assigned to lack of efficacy or another reason using this conditional proportion. Patients discontinuing treatment because of lack of efficacy returned in the following cycle to the Week 12 MMD and acute medication levels of their paired no preventive treatment patient.

For patients discontinued for other reasons, the positive incremental Week 12 MMD benefit was defined as:

$$\Delta_{i}=\max(0,{\mathrm{MMD}}_{\mathrm{no}\;\text{preventive},i}-{\mathrm{MMD}}_{\text{erenumab},i})$$

The post-discontinuation waning profile was informed by the aggregate MMD trajectory reported by Gantenbein et al. (22). The proportions of the positive Week 12 incremental benefit retained during the first, second, and third 4-week cycles after discontinuation were 0.91, 0.64, and 0.45, respectively, with no residual incremental benefit thereafter. The post-discontinuation MMD was therefore calculated as:

$${\mathrm{MMD}}_{i,s}={\mathrm{MMD}}_{\text{erenumab},i}+[1-R(s)]\Delta_{i}$$

where *i* indexes simulated patients, *s* denotes the 4-week cycle after treatment discontinuation, and *R(s)* is the retained-effect proportion in cycle *s*. Acute medication days followed the same patient-level treatment status, discontinuation reason, and retained-effect profile. Erenumab acquisition costs were applied only while the patient remained on treatment.

### 2.6 Costs

Economic inputs are summarized in Table 2. Costs included erenumab acquisition costs, acute medication costs, and migraine-related non-drug medical service costs.

**Table 2:** Economic inputs.

Parameter	Base value	Range	Source/Notes
Cost			
Erenumab acquisition cost, CNY per 70 mg	3,498.00	2,798.40–4,197.60	(32)
Daily cost of acute medication, CNY/day	31.75	28.07–36.39	(23) and Supplementary Table 2
Non-drug medical service cost, CNY per cycle	6.21	4.97–7.45	(3)
Utility			
0–3 MMD	0.71	0.60–0.82	(24)
4–9 MMD	0.61	0.50–0.72	(24)
10–14 MMD	0.57	0.46–0.68	(24)
15–19 MMD	0.50	0.40–0.60	(24)
20–23 MMD	0.48	0.38–0.58	(24)
24–28 MMD	0.38	0.28–0.48	(24)
Threshold			
1 × GDP per capita WTP, CNY/QALY	99,665.00	—	(33)
3 × GDP per capita WTP, CNY/QALY	298,995.00	—	(33)

All costs are expressed in 2025 CNY. CNY, Chinese yuan; MMD, monthly migraine day; WTP, willingness-to-pay; QALY, quality-adjusted life year.

Erenumab was priced at CNY 3,498 per 70-mg box. The base case assumed one box every 4 weeks while on treatment.

Acute medication costs were calculated by multiplying the number of acute medication days by the weighted daily medication cost. The weighted daily cost was CNY 31.75. It was derived from Chinese acute antimigraine medication category proportions and representative daily costs (23). The detailed calculation is shown in Supplementary Table 2.

The cost of non-drug medical services was CNY 6.21 per 4-week cycle. This was based on migraine-related diagnosis and non-drug medical service costs reported in the CHIRA database study, converted to CNY, and adjusted to 2025 (3).

Adverse event management costs were not included in the base case. In DRAGON, the overall safety and tolerability profile of erenumab was broadly comparable with placebo, although constipation was more frequent with erenumab (13). Given the short time horizon, the expected small cost impact, and the lack of China-specific adverse event management cost data, adverse event costs were excluded from the base-case model. This assumption is unlikely to favor the no preventive treatment arm because including adverse event management costs would primarily add to the erenumab arm.

### 2.7 Utility

Utility values were assigned by MMD health state, as shown in Table 2. As China-specific EQ-5D utility values by MMD state were not available, the model used MMD state-specific utility values from a published erenumab economic model (24, 25).

These utility inputs were selected because they were defined by MMD health states and could therefore be directly linked to the model structure. They were not China-specific, and this was addressed by varying utility values in sensitivity analyses. The use of non-China-specific utility values was also considered when interpreting QALY gains.

### 2.8 Outcomes

The main outcomes were total costs, total QALYs, incremental costs, incremental QALYs, the incremental cost-effectiveness ratio (ICER), and incremental net monetary benefit (INMB). The ICER was calculated as the incremental costs divided by the incremental QALYs.The INMB was Calculated as:

INMB=Incremental QALYs×λ−Incremental cost

where λ is the willingness-to-pay (WTP) threshold. An INMB greater than 0 indicates that erenumab preventive treatment is cost-effective at the specified threshold.

The WTP thresholds were 1× and 3× China’s Gross Domestic Product (GDP) per capita, set at CNY 99,665 and CNY 298,995 per QALY, respectively.

### 2.9 Sensitivity and scenario analyses

One-way sensitivity analysis was conducted at the WTP threshold of 3 × GDP per capita. The outcome was INMB. The analysis assessed the effect of uncertainty in key clinical, utility, cost, and discontinuation inputs.

Probabilistic sensitivity analysis used 1,000 iterations, with 10,000 simulated patients per iteration. In the probabilistic sensitivity analysis, utility parameters were sampled using beta-PERT distributions, cost parameters using gamma distributions, response and discontinuation probabilities using beta distributions, and Week 12 MMD changes using normal distributions. The cost-effectiveness acceptability curve was generated over WTP thresholds ranging from CNY 0 to CNY 300,000 per QALY.

A calibration trade-off robustness analysis was conducted to assess whether the economic results were sensitive to the secondary MMD < 15 target on the calibrated Week 12 distribution. The base-case two-component truncated normal framework, parameter bounds, primary calibration targets, and subsequent economic simulation were unchanged. The objective weight assigned to the MMD < 15 target was varied from 0 to 4.8 across prespecified values. Incremental QALYs, the ICER, and INMB were then compared across these calibrations.

A 12-week time horizon scenario analysis was conducted to examine results limited to the DRAGON double-blind period. This scenario used only the first three 4-week cycles and did not apply maintenance extrapolation.

Threshold price analysis estimated the erenumab price per 70-mg box that would make INMB equal to 0 at WTP thresholds of 1× and 3× GDP per capita.

## 3 Results

The calibration closely reproduced the primary targets. The simulated Week 12 mean MMD values were 11.005 for erenumab and 12.603 for no preventive treatment, and the simulated ≥50% MMD responder rates were 0.47079 and 0.36610, respectively. The secondary MMD < 15 target was not matched exactly, consistent with its intended role as a soft target. The simulated MMD < 15 proportions were 0.57342 and 0.47791, compared to target values of 0.523 and 0.410, respectively. Full calibration results are provided in Supplementary Table 1.

The calibration trade-off analysis showed that increasing the weight assigned to the secondary MMD < 15 target moved the calibrated MMD < 15 proportions toward the corresponding DRAGON-derived proxy values. Although the resulting Week 12 distributions varied, the primary mean MMD and responder-rate targets remained closely reproduced. Incremental QALYs and the ICER changed only modestly, and the ICER remained well above the evaluated cost-effectiveness thresholds throughout the analysis. Detailed results are shown in Supplementary Figure 3.

### 3.1 Base-case results

The base-case results are shown in Table 3. Over 52 weeks, erenumab preventive treatment generated 0.572 QALYs, and no preventive treatment generated 0.557 QALYs. The incremental QALY gain was 0.015.

**Table 3:** Base-case results.

Outcome	Erenumab preventive treatment	No preventive treatment	Incremental
Total cost, CNY	45,810.21	4,281.59	41,528.62
QALYs	0.572	0.557	0.015
ICER, CNY/QALY	—	—	2,830,387.52
INMB at 1 × GDP per capita, CNY	—	—	−40,066.30
INMB at 3 × GDP per capita, CNY	—	—	−37,141.64

CNY, Chinese yuan; QALY, quality-adjusted life year; ICER, incremental cost-effectiveness ratio; INMB, incremental net monetary benefit.

Total costs were CNY 45,810.21 for erenumab preventive treatment and CNY 4,281.59 for no preventive treatment. The incremental cost was CNY 41,528.62. The ICER was CNY 2,830,387.52 per QALY gained.

At WTP thresholds of 1× and 3× GDP per capita, INMB values were CNY − 40,066.30 and CNY − 37,141.64, respectively. These results indicate that erenumab preventive treatment was unlikely to be cost-effective at the evaluated thresholds.

### 3.2 One-way sensitivity analysis

Figure 2 shows the one-way sensitivity analysis at the WTP threshold of 3 × GDP per capita. The figure presents the 10 parameters with the largest effect on INMB. The vertical line represents the base-case INMB. All tested parameter ranges resulted in negative INMB values.

**Figure 2:**
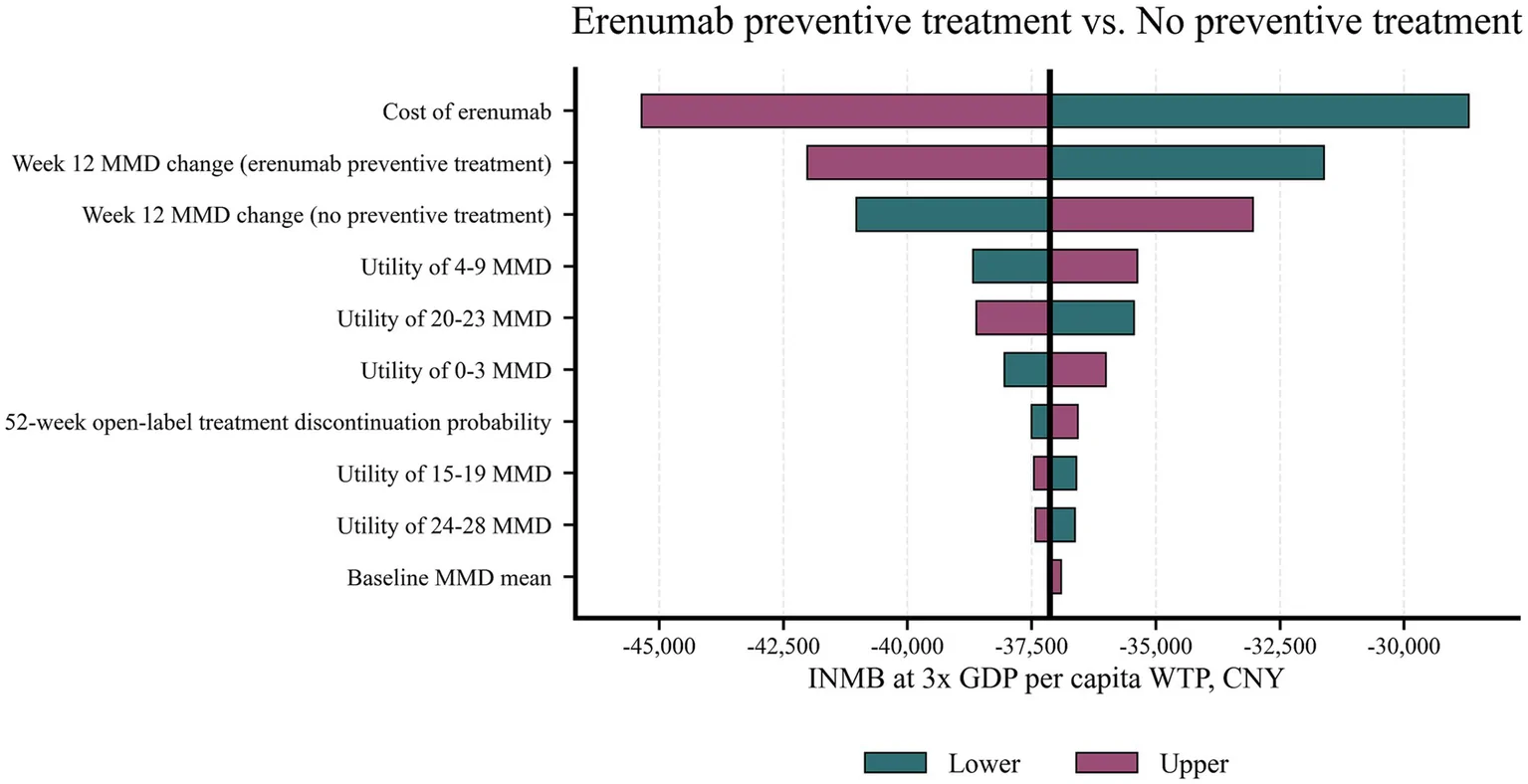
One-way sensitivity analysis at a WTP threshold of 3 × GDP per capita. The figure shows the 10 parameters with the largest effect on INMB. The vertical line represents the base-case INMB. All tested parameter ranges resulted in negative INMB values. INMB, incremental net monetary benefit; WTP, willingness-to-pay.

Erenumab acquisition costs had the largest effect on INMB. Week 12 MMD changes in the erenumab arm, Week 12 MMD changes in the no preventive treatment arm, and selected MMD state-specific utility values were also influential.

### 3.3 Probabilistic sensitivity analysis

The cost-effectiveness acceptability curve is shown in Supplementary Figure 4. In 1,000 probabilistic sensitivity analysis iterations, erenumab preventive treatment was not cost-effective at any evaluated WTP threshold ranging from CNY 0 to CNY 300,000 per QALY. No preventive treatment was cost-effective in all iterations across this WTP range.

### 3.4 Scenario analysis

The scenario analysis results are shown in Table 4. In the 12-week time horizon scenario, erenumab preventive treatment increased costs by CNY 10,383.29 and increased QALYs by 0.004. The ICER was CNY 2,466,755.48 per QALY gained. INMB values were CNY − 9,963.78 at a WTP threshold of 1 × GDP per capita and CNY − 9,124.74 at a WTP threshold of 3 × GDP per capita.

**Table 4:** Scenario analysis.

Scenario	Time horizon	Incremental cost, CNY	Incremental QALYs	ICER, CNY/QALY	INMB at 1 × GDP, CNY	INMB at 3 × GDP, CNY
Base case	52 weeks	41,528.62	0.015	2,830,387.52	−40,066.30	−37,141.64
12-week time horizon	12 weeks	10,383.29	0.004	2,466,755.48	−9,963.78	−9,124.74

QALY, quality-adjusted life year; ICER, incremental cost-effectiveness ratio; INMB, incremental net monetary benefit.

The 12-week scenario was consistent with the base case. The main conclusion did not depend only on extrapolating the maintenance period.

### 3.5 Threshold price analysis

The threshold price results are shown in Table 5 and Figure 3. At a WTP threshold of 1 × GDP per capita, the threshold price was CNY 141.82 per 70-mg box, equal to 4.05% of the current price. At a WTP threshold of 3 × GDP per capita, the threshold price was CNY 386.81 per 70-mg box, equal to 11.06% of the current price.

**Table 5:** Threshold price analysis.

WTP threshold	WTP, CNY/QALY	Threshold price, CNY/70 mg	Current price, CNY/70 mg box	Threshold/Current price
1 × GDP per capita	99,665.00	141.82	3,498.00	4.05%
3 × GDP per capita	298,995.00	386.81	3,498.00	11.06%

WTP, willingness-to-pay; CNY, Chinese yuan.

**Figure 3:**
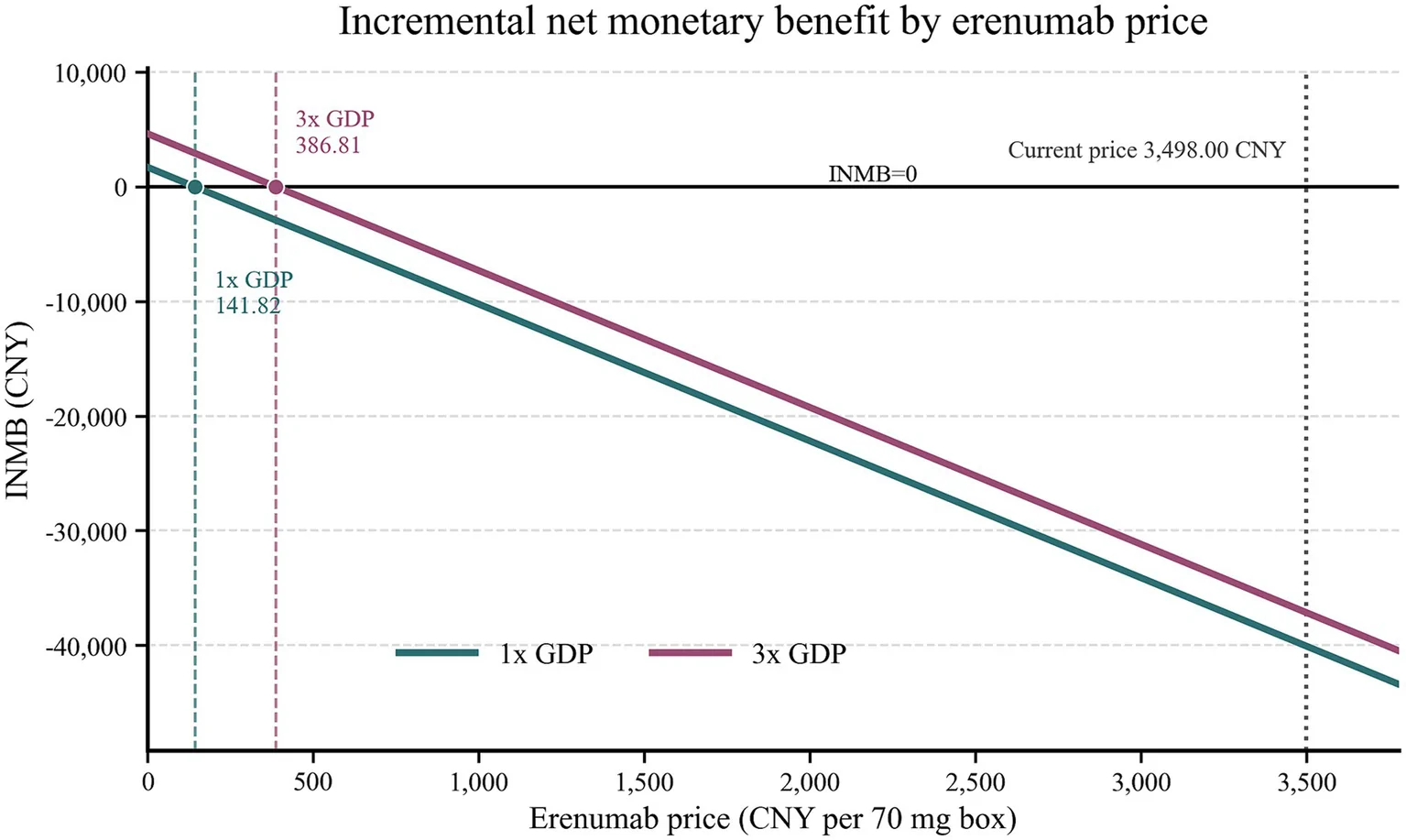
Incremental net monetary benefit across erenumab prices. The horizontal solid line represents INMB = 0. Vertical dashed lines indicate the erenumab threshold prices at WTP thresholds of 1× and 3× China’s GDP per capita. The vertical dotted line represents the current base-case erenumab price. INMB was negative at the current price at both WTP thresholds. INMB, incremental net monetary benefit; CNY, Chinese yuan.

At the current base-case price of CNY 3,498 per 70-mg box, INMB remained negative at both thresholds.

## 4 Discussion

This study evaluated erenumab preventive treatment compared to no preventive treatment for adults with chronic migraine in China. The model showed that erenumab preventive treatment increased QALYs but also substantially increased costs. At the current price, the health gain was not sufficient to offset the additional cost of the drug.

The base-case ICER was CNY 2,830,387.52 per QALY gained. INMB was negative at both 1 × and 3 × GDP per capita. One-way sensitivity analysis, probabilistic sensitivity analysis, and the 12-week scenario supported the same conclusion.

The result is mainly explained by the cost structure of the model. Erenumab preventive treatment reduced acute medication use, but the resulting cost offset was small. Most of the incremental cost came from erenumab acquisition. This is also reflected in the threshold price analysis. Even at a WTP threshold of 3 × GDP per capita, the threshold price was CNY 386.81 per 70-mg box, far below the current base-case price of CNY 3,498. Erenumab, therefore, provided clinical benefit in the model, but the benefit was not sufficient to offset its additional cost from the health system perspective.

The choice of no preventive pharmacotherapy also limits the direct applicability of the present analysis to decisions involving active preventive treatments. Recent Chinese registry data indicate that only approximately one-quarter of patients with an indication for preventive treatment are currently receiving preventive pharmacotherapy (26), suggesting that no preventive pharmacotherapy remains a clinically relevant treatment state. At the same time, flunarizine and topiramate remain among the main conventional preventive options in China (27, 28), and their acquisition costs are generally low under the current procurement and pricing environment. Comparator choice can materially affect cost-effectiveness estimates (24). Nevertheless, the direction and magnitude of the ICER change for a specific active comparator cannot be determined without a formal comparative analysis because both relative effectiveness and costs would change. The large difference between the current erenumab price and the estimated threshold price suggests that the acquisition cost of low-cost conventional oral preventives alone is unlikely to eliminate the observed economic gap; this is a qualitative interpretation rather than a cost-effectiveness result based on an active comparator. Future evaluations could use indirect treatment comparisons or network meta-analyses to estimate relative treatment effects when head-to-head evidence is unavailable. Such analyses should consider differences in patient populations, outcome definitions, assessment time points, and placebo responses across trials and combine comparative effect estimates with China-specific treatment costs, persistence, adverse events, and healthcare utilization.

The comparator arm, informed by the placebo group of DRAGON, demonstrated substantial improvement over 12 weeks. Such prominent placebo responses are well-documented in migraine prevention trials, often driven by contextual effects and regression to the mean (29). Accordingly, the placebo trajectory observed in DRAGON may not fully reproduce the clinical course of patients receiving no preventive pharmacotherapy in routine practice. If such patients would experience less improvement than the DRAGON placebo group, the present model may underestimate the incremental clinical and QALY benefit of erenumab. The magnitude of this effect, however, cannot be quantified reliably from the available data.

The present analysis was conducted from the Chinese health system perspective and therefore did not include productivity losses, patient and caregiver time, or other non-healthcare costs. This exclusion is potentially important in migraine. A national Chinese population survey found that indirect costs accounted for the majority of the economic burden of primary headache disorders, while a more recent study among bank employees in China identified work productivity loss as a major driver of societal costs and estimated employer costs to be substantially greater than direct healthcare costs (2, 30). A systematic review of economic evaluations in chronic migraine similarly found that productivity costs can exceed direct medical costs in societal perspective models, while also highlighting limited generalizability across jurisdictions (18). Previous economic evaluations have reported more favorable cost-effectiveness estimates for erenumab when productivity costs were included, although the magnitude of this effect varied across settings. A US analysis reported substantially improved economic outcomes from the societal perspective (24), whereas a Greek analysis found only a modest reduction in the ICER after incorporating work productivity loss and still required a substantial reduction in the erenumab price to meet the local cost-effectiveness threshold (31). Accordingly, the present health system analysis may underestimate the broader societal value associated with effective migraine prevention. Future societal perspective analyses should incorporate China-specific data linking MMD and treatment response to absenteeism, presenteeism, unpaid work, caregiver time, employment status, and wages.

The model has several strengths. First, the primary source of efficacy was DRAGON, an Asian trial that included adults with chronic migraine and is relevant to the target population. Second, the model used continuous MMDs as the clinical state variable, thereby linking clinical efficacy directly to utility and acute medication use. Third, the Week 12 distribution was calibrated to match key aggregate clinical outcomes because individual-level trial data were unavailable.

The study also has some limitations. First, DRAGON provided double-blind comparative evidence for 12 weeks. The 52-week base case therefore required assumptions regarding treatment continuation and the maintenance of clinical benefit after Week 12. In particular, treatment persistence observed in the 52-week open-label extension may not fully represent routine clinical practice. Selection into and continued participation in the extension study may have affected both the observed treatment discontinuation rate and the distribution of discontinuation reasons. Nevertheless, in the absence of long-term erenumab persistence data from Chinese patients, the 52-week open-label extension provides the most directly relevant empirical discontinuation experience for the same treatment and chronic migraine population and was therefore retained as the base-case source. Longer-term Chinese real-world data would improve future analyses.

Second, individual-level DRAGON data were unavailable, so continuous Week 12 MMD values had to be generated by calibration from aggregate trial endpoints. Although the calibration reproduced the primary aggregate targets, uncertainty remains regarding the distributional shape within each treatment arm. The MMD < 15 target was also used only as a soft target because it was informed by a chronic-to-episodic migraine endpoint based on headache days rather than MMDs. The calibration trade-off robustness analysis showed that varying the influence of the MMD < 15 target produced only modest changes in economic outcomes and did not alter the overall cost-effectiveness conclusion. However, because utility values are assigned according to discrete MMD states, residual uncertainty in the calibrated MMD distribution may still affect QALY estimates.

Third, utility values were derived from published international economic models rather than China-specific EQ-5D data by MMD state. These values were used because they matched the MMD health states in the present model. However, differences in population characteristics and health state valuation may affect incremental QALY estimates and, consequently, the ICER. Utility parameters were varied in both deterministic and probabilistic sensitivity analyses. INMB remained negative across all tested ranges. China-specific utility data by MMD state would nevertheless improve the transferability of future analyses.

Fourth, non-drug medical service costs were derived from migraine claims data and were not stratified by chronic migraine status or MMD severity. This may underestimate resource use among more severely ill patients. Nevertheless, this cost component was small compared to the erenumab acquisition cost.

Overall, the analysis suggests that erenumab preventive treatment may provide clinical benefit but is unlikely to be cost-effective for broad use in Chinese adults with chronic migraine at the current price. The result was mainly driven by the erenumab acquisition cost. A lower price would be required for erenumab to approach commonly used WTP thresholds in China. Targeted use in patients with a higher disease burden and response-based continuation rules may be worth exploring in future analyses, but these strategies were not formally evaluated in the present model.

## 5 Conclusion

From the Chinese health system perspective, erenumab preventive treatment for adults with chronic migraine provided modest QALY gains compared to no preventive treatment. At the current base-case price, erenumab was unlikely to be cost-effective at commonly used GDP-based WTP thresholds in China. The result was driven mainly by the drug acquisition cost. Substantial price reduction would be required for erenumab to meet these thresholds, and future analyses could further examine active comparators, longer-term real-world outcomes, and targeted treatment strategies.

## Data Availability

The original model parameters and simulated datasets presented in the study can be found in the article/Supplementary material. Furthermore, the complete microsimulation model source code, synthetic data, and Python scripts supporting the conclusions of this article can be found in the online public repository: GitHub (https://github.com/GThanatoss/erenumab-cm-china-cea-release).
