# Supplementary material for: Cost-utility analysis of erenumab preventive treatment for chronic migraine in China
Source: Front Public Health. 2026 Sep 21;14:1878335. doi: 10.3389/fpubh.2026.1878335 (PMC13634958; doi:10.3389/fpubh.2026.1878335)
Supplement: Supplementary file 1 [file Data_Sheet_1.DOCX]

Supplementary Material

**Supplementary Table 1. Week 12 calibration targets and model fit**

| **Calibration target** | **Erenumab target** | **Erenumab simulated** | **Difference** | **No preventive treatment target** | **No preventive treatment simulated** | **Difference** |
| --- | --- | --- | --- | --- | --- | --- |
| Week 12 mean MMD | 11 | 11.005 | 0.005 | 12.6 | 12.603 | 0.003 |
| Week 12 ≥50% MMD responder rate | 0.47 | 0.47079 | 0.00079 | 0.367 | 0.3661 | −0.00090 |
| Week 12 MMD < 15 | 0.523 | 0.57342 | 0.05042 | 0.41 | 0.47791 | 0.06791 |

MMD, monthly migraine days.

**Supplementary Table 2. Weighted daily cost of acute medications**

| **Medication category** | **Proportion** | **Representative drug** | **Daily cost, CNY** | **Range, CNY** | **Contribution to weighted daily cost, CNY** |
| --- | --- | --- | --- | --- | --- |
| NSAIDs | 0.2875 | Ibuprofen | 1.73 | 0.32–9.96 | 0.50 |
| Caffeine-containing medications | 0.2272 | Paracetamol/caffeine tablet | 11.36 | 2.16–16.05 | 2.58 |
| Opioids | 0.1641 | Paracetamol and dihydrocodeine tablet | 10.57 | 4.56–12.78 | 1.73 |
| Acetaminophen | 0.019 | Acetaminophen | 0.51 | 0.22–2.06 | 0.01 |
| Triptans | 0.2679 | Rizatriptan benzoate | 99.76 | 99.76–99.76 | 26.72 |
| Antiemetics | 0.0345 | Metoclopramide injection | 5.9123 | 0.2600–29.7000 | 0.20 |
| Weighted daily cost | 1 | — | — | — | 31.75 |

The weighted daily cost was calculated as the sum of medication-category proportions multiplied by representative daily costs. Lower and upper values were calculated using the same category proportions and the lower or upper representative daily costs (1). CNY, Chinese yuan; NSAIDs, non-steroidal anti-inflammatory drugs.

**Supplementary Figure 1. Simulated baseline MMD distribution.** The baseline MMD distribution was generated using a truncated normal distribution bounded between 8 and 28 days per 4 weeks. The target post-truncation mean and standard deviation were 19.2 and 5.4, respectively.

**Supplementary Figure 2. Model-calibrated Week 12 continuous distribution of monthly migraine days.** Week 12 continuous MMD distributions were generated through calibration to reproduce Week 12 mean MMD and the ≥50% MMD responder rate reported in DRAGON (2). The shape of the distribution reflects the calibration method and available aggregate targets. MMD, monthly migraine days.

**Supplementary Figure 3. Calibration trade-off robustness analysis.** The weight assigned to the secondary Week 12 MMD <15 target was varied across prespecified values while the primary calibration targets, distributional framework, and subsequent economic model were retained. Increasing the influence of the MMD <15 target moved the calibrated proportions toward the corresponding DRAGON-derived proxy values, while incremental QALYs and ICERs changed only modestly and the overall cost-effectiveness conclusion remained unchanged.

**Supplementary Figure 4.** **Cost-effectiveness acceptability curve.** The curve was generated from 1,000 probabilistic sensitivity analysis iterations. In these iterations, erenumab preventive treatment was not cost-effective at any evaluated willingness-to-pay threshold from CNY 0 to CNY 300,000 per QALY.

**References**

1.	Huang J, Wang X, Jin Y, Lou G, Yu Z. Trends and Prescribing Patterns of Antimigraine Medicines in Nine Major Cities in China from 2018 to 2022: A Retrospective Prescription Analysis. *J Headache Pain* (2024) 25(1):62. doi: 10.1186/s10194-024-01775-6.

2.	Yu S, Kim BK, Wang H, Zhou J, Wan Q, Yu T, et al. A Phase 3, Randomised, Placebo-Controlled Study of Erenumab for the Prevention of Chronic Migraine in Patients from Asia: The Dragon Study. *J Headache Pain* (2022) 23(1):146. Epub 20221121. doi: 10.1186/s10194-022-01514-9.
